# Whole-Genome Sequences of Five Geobacillus stearothermophilus Strains Isolated from Processing Lines of Powdered Infant Formula

**DOI:** 10.1128/MRA.01452-18

**Published:** 2019-01-24

**Authors:** Tae Jin Cho, Dongmin Yang, Byeonghyeok Park, In-Geol Choi, Min Suk Rhee

**Affiliations:** a Department of Biotechnology, College of Life Sciences and Biotechnology, Korea University, Seoul, South Korea; University of Delaware

## Abstract

Geobacillus stearothermophilus is the thermophile present in processing lines of powdered infant formula (PIF). We report the whole-genome sequences of G. stearothermophilus strains isolated from work-in-process products (sterilized and concentrated milk) of manufacturing plants. Understanding the genomic basis governing the metabolism of G. stearothermophilus can contribute to the safety management of PIF during its manufacture.

## ANNOUNCEMENT

Geobacillus stearothermophilus is a Gram-positive spore-forming thermophile ubiquitously present in powdered infant formula (PIF) processing plants (1, 2). The general processing steps of PIF consist of the blending of raw ingredients, heat sterilization, condensation, and spray drying (3). Since the high heat resistance of G. stearothermophilus spores means the bacterium can endure the heat treatment processes during the manufacture of PIF, growth of G. stearothermophilus in poststerilization steps has been regarded to be the major cause of the production of spoilage metabolites (4, 5). To understand the metabolic pathways and major determinants for metabolism which result in the deterioration of PIF products, functional genes governing key metabolisms should be identified by genomic analysis.

We sequenced five strains of G. stearothermophilus obtained from the work-in-process products (WIP) of PIF processing lines through the following isolation method: WIP samples (25 ml of concentrated or pasteurized milk) (Table 1) were homogenized with 225 ml of sterile 0.85% (wt/vol) saline using a stomacher (Circulator 400, Seward) at 230 rpm for 2 min. One milliliter of homogenized sample was added in 0.85% saline for serial 10-fold dilutions, and 100 μl of diluted sample was spread plated on plate count agar (PCA; Difco, Sparks, MD, USA), followed by incubation at 55°C for 48 h. Single colonies on PCA were substreaked onto tryptic soy agar (TSA; Difco, Becton, Dickinson), and the TSA was incubated, followed by the propagation of a single colony on those plates in 5 ml tryptic soy broth (TSB; Difco) at 55°C for 48 h. Bacterial suspensions were mixed with a 50% glycerol solution to prepare stock cultures and stored at −80°C.

**TABLE 1:** Sequencing and genome features of *Geobacillus stearothermophilus* strains

	Data for strain:				
Feature	FHS-PHGT51	FHS-PCGT429	FHS-PPGT111	FHS-PPGT130	FHS-PCGT134
Origin	Concentrated milk	Concentrated milk	Pasteurized milk	Pasteurized milk	Concentrated milk
GenBank accession no.	RCTG00000000	RCTH00000000	RCTI00000000	RCTJ00000000	RCTK00000000
BioSample no.	SAMN10227540	SAMN10227541	SAMN10227542	SAMN10227544	SAMN10227543
BioProject no.	PRJNA496302	PRJNA496303	PRJNA496304	PRJNA496305	PRJNA496306
SRA accession no.	SRR8074255	SRR8074312	SRR8074389	SRR8074739	SRR8080672
Total no. of sequencing reads (paired ends) (seqs)	2,284,199	2,189,920	1,487,742	2,651,209	2,642,957
Total read length (Mbp)	1,266	1,191	815	1,507	1,445
No. of quality-controlled reads (paired ends) (seqs)	2,267,818	2,176,727	1,475,308	2,638,836	2,625,725
Quality-controlled read length (Mbp)	963	850	602	1,119	1,104
Avg insert size (bp)	322.5	318.1	318.1	308.9	314.1
Total length of assembled sequence (bp)	2,894,956	2,667,663	2,886,901	2,871,168	2,879,781
Sequencing read coverage depth (fold)	746.72	723.74	470.42	875.59	861.33
No. of contigs	330	150	328	328	298
Contig *N*_50_ (bp)	34,230	68,942	33,321	36,149	34,944
Contig *L*_50_	25	12	24	22	22
Maximum contig length (bp)	167,255	204,326	256,326	256,473	256,423
Overall GC content (%)	52.30	52.94	52.13	52.20	52.18

Each stock culture (50 μl) was incubated in 4.95 ml TSB at 55°C for 24 h. The enriched culture was streaked onto TSA and propagated at 55°C for 24 h. Then, a single colony from a substreaked TSA plate was inoculated in 5 ml TSB for incubation at 55°C, and the enriched culture with a cell density of ca. 9-log CFU/ml (incubation time was set by the preliminary tests) was used for the extraction of genomic DNA (gDNA). The DNeasy blood and tissue kit (Qiagen, Valencia, CA) was utilized to extract gDNA from a 1-ml aliquot of bacterial culture after the pretreatment specific for Gram-positive bacteria according to the manufacturer’s instructions.

A library for whole-genome sequencing was prepared with a Nextera DNA library prep kit (Illumina, Inc., San Diego, CA, USA). Sequencing was carried out with 600 cycles of reads by the Illumina MiSeq platform. An average 2,251,205 paired-end reads with a read size of 301 × 2 bp were obtained (Table 1). Trimming of the adapter sequences and low-quality bases (<Q20) from raw reads was performed using the Trim Galore! software (version 0.4.4; https://www.bioinformatics.babraham.ac.uk/projects/trim_galore/). An average 2,236,883 reads per strain could pass the quality control and were assembled using the SPAdes genome assembler (version 3.12.0) (6). The assemblies were annotated preliminarily using the Prokaryotic Contigs Annotation Pipeline Server (P-CAPS, version 0.1) with default parameters (7). The final annotation was generated with the NCBI Prokaryotic Genome Annotation Pipeline (PGAP, version 4.6) with default parameters (8).

A summary of the characteristics and genome features of the five strains of G. stearothermophilus is presented in Table 1. The general statistics for the genomes were as follows: sequencing reads in total (paired ends), 1,487,742 to 2,651,209 sequences (seqs); total read length, 815 to 1,507 Mbp; total length of assembled sequence, 2,667,663 to 2,894,956 bp; number of contigs, 150 to 330; overall GC content, 52.13 to 52.94%; and coverage depth, 470.42- to 875.59-fold.

The whole-genome sequence information reported here is expected to broaden our knowledge regarding the genetic and functional characteristics of G. stearothermophilus with regard to the production of metabolites during the manufacture of PIF.

### Data availability.

All genome sequences and raw reads reported here were deposited in GenBank under the accession, BioSample, and BioProject numbers listed in Table 1. The versions described in this paper are the first versions.
